# Supplementary material for: Prevalence and risk factors for latent tuberculosis infection among household contacts of index cases in two South African provinces: Analysis of baseline data from a cluster-randomised trial
Source: PLoS One. 2020 Mar 17;15(3):e0230376. doi: 10.1371/journal.pone.0230376 (PMC7077873; doi:10.1371/journal.pone.0230376)
Supplement: S1 Table — Putative causal pathways are shown by arrowed lines. Unmeasured variables are filled in white. Using the backdoor criteria, study site, household contact sex and HIV status, and index case age, sex, HIV status and microbiological TB status were identified as the minimal sufficient adjustment set of covariates for estimating the direct effect of index case HIV status on latent TB infection. (DOCX) [file pone.0230376.s001.docx]

**Supplemental Table 1: Characteristics of household contacts who did and did not receive tuberculin skin testing**

|  | **TST done (N=2725)** | **TST not done (N=260)** |
| --- | --- | --- |
| **Site** |  |  |
| Mangaung | 1481 (87.8%) | 206 (12.2%) |
| Capricorn | 1244 (95.8%) | 54 (4.2%) |
| **Sex** |  |  |
| Female | 1694 (91.7%) | 154 (8.3%) |
| Male | 1031 (90.7%) | 106 (9.3%) |
| **Age (years, median, IQR)** | 16.0 (8.0, 37.0) | 21.0 (4.0, 37.0) |
| **Time spent with index TB case** |  |  |
| Every now and again | 345 (94.8%) | 19 (5.2%) |
| Part of the day | 1310 (93.5%) | 91 (6.5%) |
| Most of the day | 1068 (87.8%) | 149 (12.2%) |
| **Slept in same bed as index TB case** |  |  |
| No | 2689 (91.2%) | 260 (8.8%) |
| Yes | 36 (100.0%) | 0 (0.0%) |
| **Shared bedroom with index TB case** |  |  |
| No | 2296 (92.3%) | 191 (7.7%) |
| Yes | 429 (86.1%) | 69 (13.9%) |
| **Smoking status** |  |  |
| Never smoked | 2465 (91.4%) | 233 (8.6%) |
| Current smoker | 216 (89.3%) | 26 (10.7%) |
| Previous smoker | 44 (97.8%) | 1 (2.2%) |
| **Alcohol use** |  |  |
| No | 2406 (91.3%) | 229 (8.7%) |
| Yes | 319 (91.1%) | 31 (8.9%) |
| **HIV status** |  |  |
| HIV negative | 2335 (94.0%) | 150 (6.0%) |
| HIV positive | 293 (84.0%) | 56 (16.0%) |
| HIV unknown | 94 (63.5%) | 54 (36.5%) |
| **ART status (if HIV-positive)** |  |  |
| Not taking ART | 35 (81.4%) | 8 (18.6%) |
| Taking ART | 207 (73.4%) | 75 (26.6%) |
