# Supplementary figures and images for: Prevalence and risk factors for latent tuberculosis infection among household contacts of index cases in two South African provinces: Analysis of baseline data from a cluster-randomised trial
Source: PLoS One. 2020 Mar 17;15(3):e0230376. doi: 10.1371/journal.pone.0230376 (PMC7077873; doi:10.1371/journal.pone.0230376)

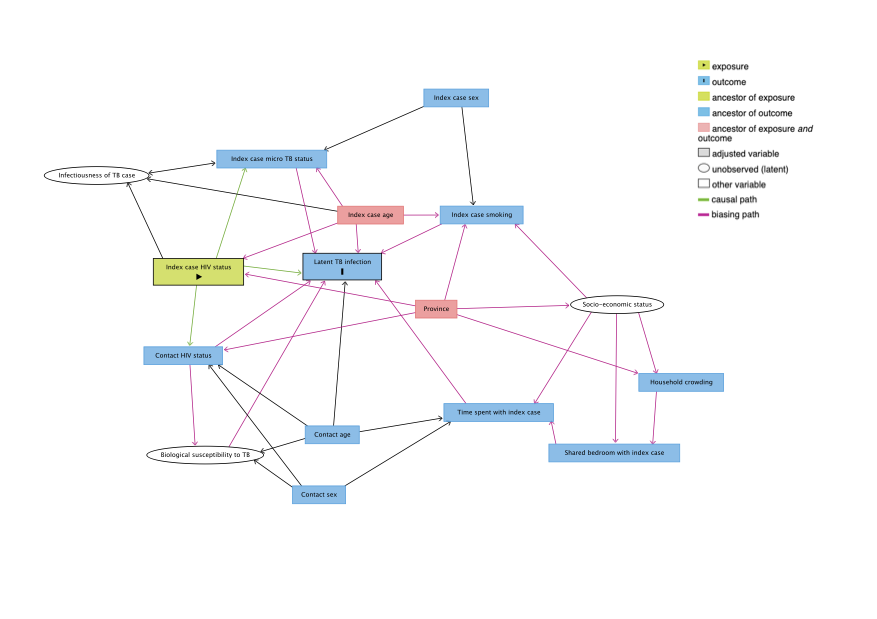

Supplement: S1 Fig — (TIFF) [file pone.0230376.s002.tiff]
